# Prevalence and Factors Associated With Symptom Profiles of Disorders of Gut‐Brain Interaction in Obesity Before and After Treatment

**DOI:** 10.1111/nmo.70017

**Published:** 2025-03-10

**Authors:** Esther Colomier, Janita Halminen, Malin Björck, Gudrún Höskuldsdóttir, Karin Mossberg, My Engström, Björn Eliasson, Ville Wallenius, Lars Fändriks, Jan Tack, Hans Törnblom, Magnus Simrén

**Affiliations:** ^1^ Department of Molecular and Clinical Medicine, Institute of Medicine, Sahlgrenska Academy University of Gothenburg Gothenburg Sweden; ^2^ Translational Research Center for Gastrointestinal Disorders (TARGID), Department of Chronic Diseases and Metabolism (CHROMETA) KU Leuven Leuven Belgium; ^3^ Department of Medicine Sahlgrenska University Hospital Gothenburg Gothenburg Sweden; ^4^ Department of Public Health and Community Medicine, Institute of Medicine, Sahlgrenska Academy University of Gothenburg Gothenburg Sweden; ^5^ Institute of Health and Care Sciences, Sahlgrenska Academy University of Gothenburg Gothenburg Sweden; ^6^ Region Västra Götaland Sahlgrenska University Hospital, Department of Surgery Gothenburg Sweden; ^7^ Department of Surgery Sahlgrenska University Hospital Gothenburg Sweden; ^8^ Center for Functional GI and Motility Disorders University of North Carolina‐Chapel Hill Chapel Hill North Carolina USA

**Keywords:** disorders of gut‐brain interaction, functional dyspepsia, irritable bowel syndrome, obesity, prevalence

## Abstract

**Background & Aims:**

Disorders of gut‐brain interaction (DGBI) in obesity could impair health outcomes. Therefore, we aimed to study the prevalence and burden of symptoms compatible with a DGBI in obesity and assess the effect of obesity treatment on comorbid DGBI.

**Methods:**

We used baseline and two‐year follow‐up data from a prospective non‐randomized cohort study including patients with obesity referred for obesity treatment. Patients completed the Rome III questionnaire before and after receiving Roux‐en‐Y gastric bypass (RYGB), sleeve gastrectomy (SG), or medical treatment. Validated questionnaires and blood parameters were used to assess the burden of DGBI in obesity.

**Results:**

In total, 939 patients (73% female, 44 ± 13 years, 42 ± 5 kg/m^2^, 36% medical treatment, 38% RYGB, 20% SG) completed the Rome III questionnaire at baseline and 651 patients (32 ± 6 kg/m^2^) at follow‐up. The proportion of patients with a DGBI symptom profile was reduced from 61% (24% esophageal, 27% gastroduodenal, 38% bowel, and 8% anorectal disorders) to 53% (15% esophageal, 25% gastroduodenal, 34% bowel, 8% anorectal disorders) at follow‐up. There was a substantial shift between the baseline and follow‐up DGBI symptom profiles across all GI regions. Patients with a DGBI symptom profile at baseline presented with more severe psychological distress, a poorer quality of life, and were more likely to be female.

**Conclusions:**

DGBI symptom profiles are common and can impair health outcomes in obesity. Obesity treatment lowers the prevalence of DGBI symptoms in general, but an important shift between baseline and follow‐up DGBI symptom profiles across all GI regions can be observed.

## Graphical abstract

DGBI symptoms are common in obesity and have the potential to exacerbate negative health outcomes. In general, the prevalence of DGBI symptoms decreases after obesity treatment, but patients can also shift from one GI symptom profile to another.

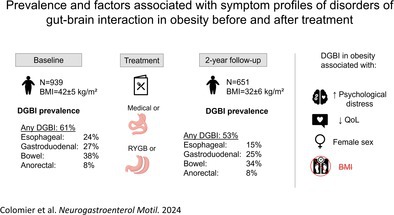

**Summary**

- Comorbid disorders of gut‐brain interaction (DGBI) in obesity have the potential to exacerbate negative health outcomes. The prevalence and burden of DGBI in obesity are understudied.
- DGBI symptom profiles across the complete gastrointestinal tract are common in obesity. In general, the prevalence of DGBI symptoms decreases after obesity treatment, but patients can also shift from one symptom profile to another.
- DGBI symptom profiles in obesity should be monitored and treated according to DGBI clinical recommendations since they have the potential to impair health outcomes, including the quality of life and psychological state of patients.

## 1 Introduction

Obesity, a condition characterized by excessive fat accumulation, is defined by a body mass index (BMI) of 30 kg/m^2^ or higher and is a consequence of an imbalance between calorie intake and expenditure influenced by genetic, environmental, and behavioral factors [1].

Previous studies have shown associations between obesity and gastrointestinal (GI) symptoms, chronically experienced by patients with disorders of gut‐brain interaction (DGBI). Patients with a DGBI have a typical GI symptom pattern, in the absence of organic diseases that could explain the symptoms, which should be excluded by a minimal relevant clinical evaluation [2]. A meta‐analysis showed that the predominant GI symptoms associated with increasing BMI are abdominal pain, gastroesophageal reflux, vomiting, chest pain or heartburn, retching, and incomplete evacuation [3, 4]. Research exploring the connection between obesity and specific gastrointestinal conditions, such as gastroesophageal reflux disease (GERD), has consistently demonstrated a positive association [5]. However, when it comes to examining the association between increasing BMI and particular DGBI, a more complex picture emerges. A multitude of studies in this area have produced conflicting outcomes. Some of these findings have, for example, described a positive correlation between irritable bowel syndrome, functional diarrhea, and increasing BMI, either overall or specific subgroups [6, 7]. In contrast, other studies have indicated the absence of any significant correlation or, intriguingly, even an inverse relationship [8, 9, 10, 11, 12, 13]. Nonetheless, there has not been an evaluation of the occurrence of various esophageal, gastroduodenal, bowel, and anorectal DGBI among individuals with obesity. Moreover, the effects of obesity treatment on the presence of concurrent DGBI have received limited research attention.

Hence, the primary objective of this study was to determine the prevalence of GI symptoms compatible with one or more of the specified DGBI in patients with obesity. In addition, we aimed to examine the factors associated with obesity and comorbid DGBI symptom profiles and to explore how various obesity treatments might impact the prevalence of DGBI symptom profiles.

## 2 Materials and Methods

### 2.1 Participants

We included data of patients from the BAriatric surgery SUbstitution and Nutrition (BASUN) study, who completed the Rome III diagnostic questionnaire at baseline [14]. The BASUN study design has been described in detail elsewhere [15]. Briefly, BASUN is a prospective non‐randomized cohort study that recruited 1127 adults with BMI ≥ 35 kg/m^2^ referred for medical or surgical obesity treatment. The patients could be categorized into three groups: class I (30–34.9 kg/m^2^), class II (35–39.9 kg/m^2^), and class III obesity (≥ 40 kg/m^2^) [16]. Only a few study participants qualified as having class I obesity, possibly because they initiated weight loss before receiving obesity treatment but after study inclusion. Study measurements were obtained at baseline, two‐year follow‐up, and are further planned at five and ten‐year follow‐up. The Ethical Regional Board of Gothenburg approved the protocol (Dr. no 673–14) and all patients provided written and verbal informed consent before the study start.

### 2.2 Obesity Treatment

The different treatments in the BASUN study were medical treatment, Roux‐en‐Y gastric bypass (RYGB) [17], and sleeve gastrectomy (SG) [18], and have been extensively discussed elsewhere [15]. In short, patients with a BMI > 35 kg/m^2^ not willing or unqualified for RYGB or SG were offered medical treatment including a very‐low energy diet (daily intake: 450–800 kcal) for 12–20 weeks. This was followed by a 12‐week reintroduction period during which a monthly replacement of a very‐low energy meal was introduced (meal intake: 300–475 kcal). Patients then continued with a personal energy‐restricted diet based on the Nordic Nutrition Recommendations (daily intake: 1400–1600 kcal) [19]. After 6 and 12 months, patients could also receive add‐on glucagon‐like peptide‐1 receptor agonists, sodium‐glucose transport protein‐2 inhibitors, orlistat, or a combination of bupropion and naltrexone when appropriate. Surgery was recommended to patients with a BMI > 30 kg/m^2^ with obesity‐related comorbidities such as diabetes and sleep apnea, or BMI > 40 kg/m^2^ without comorbidities [20, 21]. Contraindications for surgical treatment included drug or alcohol abuse, unstable psychiatric disorders, cancer during the last 5 years, or poor general health condition. Age > 60 years was a relative contraindication. For SG, occasional proton pump inhibitor use was an exclusion criterion. All patients in the BASUN study also received lifestyle advice (diet and physical activity) according to guidelines used in clinical practice.

### 2.3 Questionnaires and Metabolic Function

To identify patients with a DGBI symptom pattern across various anatomical GI regions before and after obesity treatment, we used the Rome III Diagnostic Questionnaire [14]. According to Rome Foundation recommendations, patients reporting celiac disease, GI cancer, inflammatory bowel disease or other known organic or structural conditions that could explain GI symptoms should be excluded from all DGBI diagnoses. Hence, patients were asked to report the presence of any comorbidities at baseline and were excluded accordingly. However, diagnostic tests were not performed on a regular basis, leading us to utilize the term “DGBI symptom pattern” instead of “DGBI diagnosis”.

The EuroQol five‐dimensional questionnaire (EQ‐5D) assessed current health‐related quality of life (QoL) [22]. EQ‐5D includes subscales (mobility, self‐care, everyday activities, pain/discomfort, and anxiety/depression) which are transformed into a summarizing index score from zero (a state as bad as being dead) to one (full health).

The Beck Anxiety Inventory (BAI) questionnaire was used to evaluate the frequency and severity of common anxiety symptoms (e.g., hands trembling) during a 7‐day recall period [23]. Total scores range from zero to 63, with high scores representing more severe anxiety.

The Patient Health Questionnaire‐9 (PHQ‐9) assessed depression levels (e.g., the level of interest in activities) in the last 2 weeks [24]. Total scores range from zero to 27, with high scores representing more severe depression.

At baseline, plasma glucose levels and hemoglobin A1c were measured on study‐specific blood samples. Most recent information about blood lipids (high‐density lipoprotein and low‐density lipoprotein cholesterol, triglycerides) was retrieved from medical records.

### 2.4 Data Analysis

For the descriptive analyses, including patient characteristics and DGBI symptom profile at baseline, categorical variables were described using percentages and a 95% confidence interval. Continuous variables were reported as a mean and standard deviation or a median and interquartile range.

We first compared patients with vs. without symptoms compatible with a DGBI at baseline using independent sample *t*‐tests or the Mann–Whitney *U* test. Secondly, we determined the effect of having obesity and comorbid DGBI symptom patterns across multiple GI regions using analysis of variance with linear contrast analysis. Effect sizes were demonstrated as partial eta squared (η^2^) (small to medium effects: η^2^ = 0.047–0.11, large effects: η^2^ > 0.11) [25].

Lastly, we described the proportions of patients with and without a DGBI symptom profile at baseline and at the two‐year follow‐up to assess the potential effect of obesity treatment on the prevalence of DGBI symptom patterns. Patients who did not receive treatment or who were lost to follow‐up were removed from this comparative descriptive analysis.

Supplementary tables contain information on the cohort, including only patients with diabetes (*n* = 144). These findings were reported separately to explore the role of diabetes in obesity with comorbid DGBI since diabetes could potentially account for DGBI symptom patterns as well.

Statistical analyses were performed using SPSS version 29.0.0 software (SPSS, Chicago, IL, USA), with significance set at a *p* < 0.05. All authors had access to the study data and reviewed and approved the final manuscript.

## 3 Results

### 3.1 Participants

**FIGURE 1:**
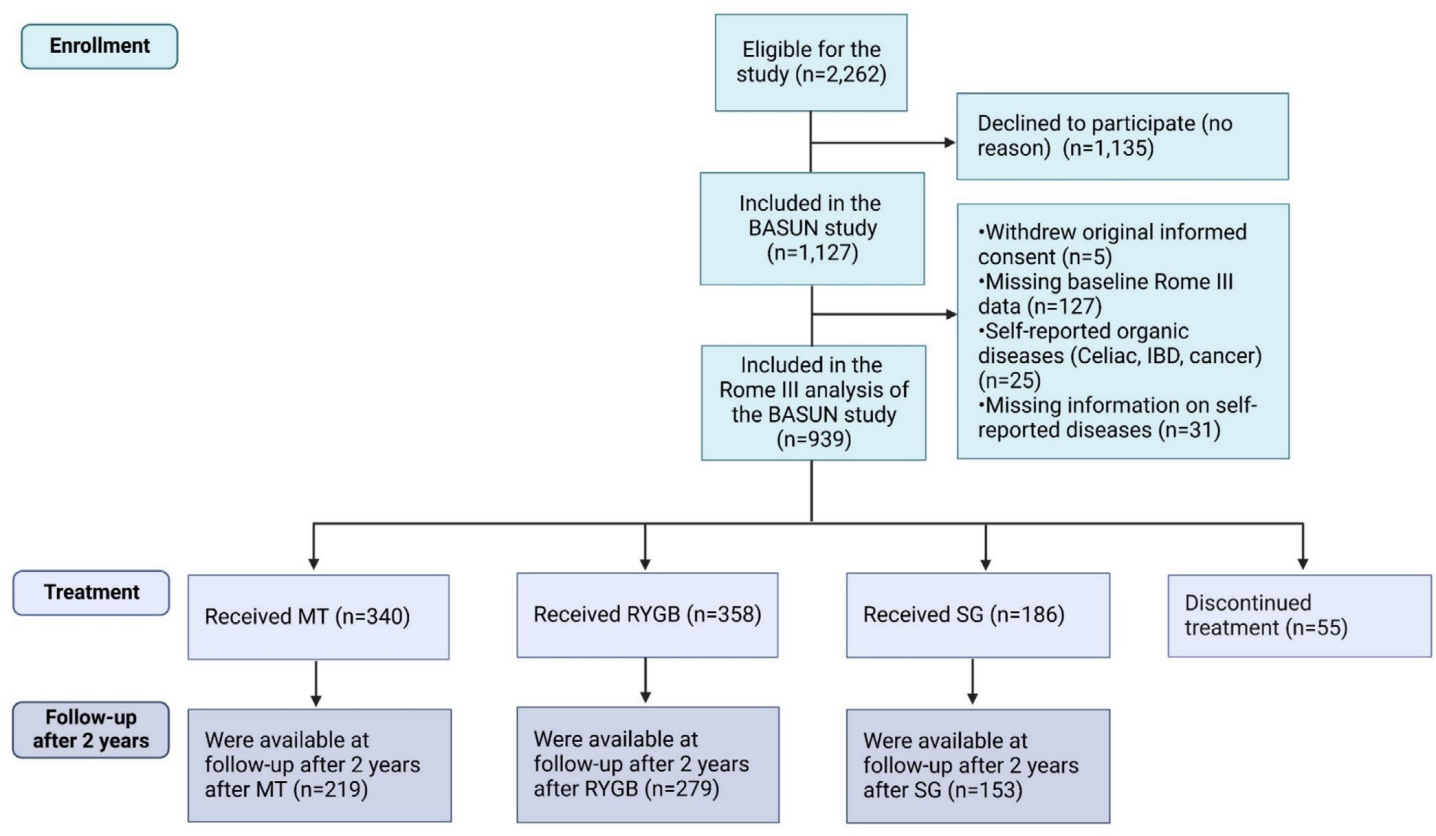
Flow chart of the study sample. IBD, INFLAMMATORY bowel disease; MT, Medical treatment; RYGB, Roux‐en‐Y gastric bypass; SG, Sleeve gastrectomy.

In total, 939 patients were included (*N* = 340 medical treatment, *N* = 358 RYGB, *N* = 186 SG, and *N* = 55 dropped out after baseline) (Figure 1). A 3:1 ratio of females to males could be observed. Most patients were between 45 and 59 years old (age: 44 ± 13 years) and could be categorized as class III obesity (BMI: 42.9 ± 4.8 kg/m^2^) (Table 1).

**TABLE 1:** Demographic factors of the BASUN cohort stratified by different treatments at baseline.

Demographic (%)	Baseline cohort stratified				
	Overall (*n* = 939)	MT (*n* = 340)	RYGB (*n* = 358)	SG (*n* = 186)	Discontinued (*n* = 55)
Gender					
Female	74.5 (71.6, 77.3)	71.8 (66.7, 76.5)	78.5 (73.9, 82.6)	75.8 (69.0, 81.8)	61.8 (47.7, 74.6)
Male	25.2 (22.5, 28.1)	27.6 (23.0, 32.7)	21.5 (17.4, 26.1)	24.2 (18.2, 31.0)	38.2 (25.4, 52.3)
Other	0.2 (0.0, 0.8)	0.6 (0.1, 2.1)	0.0 (0.0, 1.0)	0.0 (0.0, 2.0)	0.0 (0.0, 6.5)
Age groups (years)					
18–29	15.0 (12.8, 17.5)	12.1 (8.8, 16.0)	16.2 (12.5, 20.4)	18.3 (13.0, 24.6)	14.8 (6.6, 27.1)
30–44	32.6 (29.6, 35.7)	24.4 (19.9, 29.3)	35.5 (30.5, 40.7)	41.9 (34.8, 49.4)	33.3 (21.1, 47.5)
45–59	41.7 (38.5, 44.9)	41.2 (35.9, 46.6)	44.7 (39.5, 50.0)	37.6 (30.7, 45.0)	38.9 (25.9, 53.1)
60–74	10.0 (8.2, 12.1)	20.6 (16.4, 25.3)	3.6 (1.9, 6.1)	2.2 (0.6, 5.4)	13.0 (5.4, 24.9)
≥ 75	0.6 (0.2, 1.4)	1.8 (0.7, 3.8)	0.0 (0.0, 1.0)	0.0 (0.0, 2.0)	0.0 (0.0, 6.6)
BMI groups (kg/m^2^)					
Obese I^a^	2.2 (1.3, 3.3)	4.7 (2.7, 7.5)	0.6 (0.1, 2.0)	0.5 (0.0, 3.0)	2.4 (0.1, 12.6)
Obese II^b^	31.7 (28.8, 34.9)	42.9 (37.6, 48.4)	22.1 (17.9, 26.7)	25.3 (19.2, 32.1)	52.4 (36.4, 68.0)
Obese III^c^	66.1 (62.9, 69.1)	52.4 (46.9, 57.8)	77.4 (72.7, 81.6)	74.2 (67.3, 80.3)	45.2 (29.8, 61.3)
Social status					
Married	40.0 (36.9, 43.3)	39.7 (34.5, 45.1)	38.5 (33.5, 43.8)	43.0 (35.8, 50.5)	41.8 (28.7, 55.9)
Lives with parent(s)	0.3 (0.1, 0.9)	0.6 (0.1, 2.1)	0.3 (0.0, 1.5)	0.0 (0.0, 0.0)	0.0 (0.0, 6.5)
Cohabiting	24.9 (22.2, 27.8)	20.0 (15.9, 24.7)	30.4 (25.7, 35.5)	25.8 (19.7, 32.7)	16.4 (7.8, 28.8)
Single	28.4 (25.6, 31.4)	31.2 (26.3, 36.4)	26.5 (22.0, 31.4)	26.3 (20.2, 33.3)	30.9 (19.1, 44.8)
Education					
Primary	12.7 (10.6, 15.1)	15.7 (11.8, 20.3)	12.2 (8.9, 16.1)	8.7 (4.9, 13.9)	11.8 (4.4, 23.9)
Secondary	52.2 (48.8, 55.6)	44.3 (38.6, 50.0)	57.9 (52.4, 63.2)	54.3 (46.6, 61.9)	54.9 (40.3, 68.9)
Tertiary	35.1 (31.9, 38.4)	40.0 (34.5, 45.7)	30.0 (25.1, 35.2)	37.0 (29.8, 44.7)	33.3 (20.8, 47.9)

*Note:* The table displays column percentages.

Abbreviations: MT, medical treatment; RYGB, Roux‐en‐Y gastric bypass; SG, sleeve gastrectomy.

^a^ 30 kg/m^2^ < BMI < 34.9 kg/m^2^.

^b^ 35 kg/m^2^ < BMI < 39.9 kg/m^2^.

^c^ BMI > 40 kg/m^2^.

### 3.2 Prevalence of Comorbid DGBI Symptom Patterns

In total, 61% of the overall cohort and 58% of the diabetes subgroup reported DGBI symptom patterns. The patients predominantly presented with bowel, followed by gastroduodenal, esophageal, and anorectal disorders (Table 2). IBS (21%), nausea and vomiting disorders (17%), and functional heartburn (14%) were the most common DGBI. Similar observations were noted in the cohort stratified according to treatment arm apart from the prevalence of functional heartburn (8%) being slightly lower in the SG treatment arm and the prevalence of fecal incontinence (16%) slightly higher in the arm that discontinued.

**TABLE 2:** Prevalence of symptoms compatible with DGBI before obesity treatment stratified by different treatments.

Diagnosis	Overall (*n* = 939)	MT (*n* = 340)	RYGB (*n* = 358)	SG (*n* = 186)	Discontinued (*n* = 55)	
Esophageal disorders	23.3 (20.7, 26.2)	24.7 (20.2, 29.6)	22.6 (18.4, 27.3)	21.0 (15.4, 27.5)	27.3 (16.1, 41.0)	
Functional chest pain	5.6 (4.2, 7.3)	5.7 (3.5, 8.8)	4.8 (2.8, 7.6)	7.6 (4.2, 12.4)	3.7 (0.5, 12.7)	
Functional heartburn	13.7 (11.6, 16.1)	13.9 (10.4, 18.1)	16.1 (12.4, 20.3)	8.1 (4.6, 13.0)	16.4 (7.8, 28.8)	
Globus	2.7 (1.7, 4.0)	2.5 (1.1, 4.9)	2.2 (0.9, 4.4)	3.4 (1.2, 7.2)	4.0 (0.5, 13.7)	
Functional dysphagia	3.9 (2.7, 5.3)	5.1 (3.0, 8.0)	2.3 (0.9, 4.4)	4.3 (1.9, 8.3)	5.5 (1.1, 15.1)	
Gastroduodenal disorders	26.7 (23.9, 29.7)	26.5 (21.9, 31.5)	28.8 (24.1, 33.8)	23.7 (17.7, 30.4)	25.5 (14.7, 39.0)	
Functional dyspepsia	9.2 (7.4, 11.2)	11.9 (8.7, 15.9)	7.4 (4.9, 10.7)	7.6 (4.2, 12.4)	9.1 (3.0, 20.0)	
Belching disorder	7.9 (6.3, 9.9)	7.7 (5.1, 11.0)	7.9 (5.3, 11.2)	8.6 (5.0, 13.7)	7.3 (2.0, 17.6)	
Nausea and vomiting disorders	16.7 (14.4, 19.3)	15.3 (11.6, 19.6)	20.4 (16.3, 24.9)	13.4 (8.9, 19.2)	12.7 (5.3, 24.5)	
Rumination syndrome	0.6 (0.2, 1.4)	1.2 (0.3, 3.0)	0.3 (0.0, 1.6)	0.0 (0.0, 2.0)	1.9 (0.0, 9.9)	
Bowel disorders	37.7 (34.6, 40.9)	37.6 (32.5, 43.0)	35.0 (30.1, 40.2)	39.8 (32.7, 47.2)	49.1 (35.4, 62.9)	
Irritable bowel syndrome	20.5 (17.9, 23.2)	23.7 (19.2, 28.6)	15.6 (11.1, 18.8)	23.4 (17.5, 30.2)	29.1 (17.6, 42.9)	
IBS‐C	2.9 (1.9, 4.2)	3.4 (1.7, 5.9)	2.0 (0.8, 4.1)	3.4 (1.2, 7.2)	3.7 (0.5, 12.7)	
IBS‐D	5.6 (4.2, 7.3)	7.0 (4.5, 10.3)	4.9 (2.9, 7.7)	3.9 (1.6, 7.9)	7.4 (2.1, 17.9)	
IBS‐M	10.7 (8.7, 12.8)	11.9 (8.6, 15.9)	7.2 (4.7, 10.4)	13.4 (8.8, 19.3)	16.7 (7.9, 29.3)	
IBS‐U	1.2 (0.6, 2.2)	1.8 (0.7, 3.9)	0.6 (0.1, 2.1)	1.7 (0.3, 4.8)	0.0 (0.0, 6.6)	
Functional constipation	10.6 (8.7, 12.7)	8.8 (6.0, 12.4)	12.3 (9.1, 16.2)	10.8 (6.7, 16.1)	9.1 (3.0, 20.0)	
Functional diarrhea	4.3 (3.1, 5.8)	2.7 (1.2, 5.0)	5.4 (3.3, 8.2)	4.3 (1.9, 8.4)	7.3 (2.0, 17.6)	
Functional abdominal bloating/distention	5.7 (3.9, 8.0)	5.7 (2.9, 9.9)	6.1 (3.2, 10.4)	4.8 (1.6, 10.8)	6.1 (0.7, 20.2)	
Anorectal disorders	9.2 (7.4, 11.2)	12.4 (9.1, 16.4)	6.7 (4.4, 9.9)	6.0 (3.0, 10.4)	16.4 (7.8, 28.8)	
Fecal incontinence	8.8 (7.1, 10.8)	11.9 (8.6, 15.8)	6.7 (4.4, 9.9)	4.9 (2.3, 9.1)	16.4 (7.8, 28.8)	
Functional anorectal pain	0.6 (0.2, 1.4)	1.2 (0.3, 3.0)	0.0 (0.0, 1.0)	1.1 (0.1, 3.9)	0.0 (0.0, 6.6)	

Abbreviations: IBS‐C, irritable bowel syndrome with predominant constipation; IBS‐D, irritable bowel syndrome with predominant diarrhea; IBS‐M, irritable bowel syndrome with mixed bowel habits; IBS‐U, irritable bowel syndrome unsubtyped; MT, medical treatment; RYGB, Roux‐en‐Y gastric bypass; SG, sleeve gastrectomy.

### 3.3 Factors Associated With Comorbid DGBI Symptom Patterns

Patients with DGBI symptom patterns contained a higher proportion of females (79% vs. 68%, *p* < 0.001), reported more severe anxiety (BAI: 10 [14] vs. 5 [8], *p* < 0.001) and depression (PHQ‐9: 7 [9] vs. 4 [6], *p* < 0.001), and poorer QoL (EQ5D: 0.64 ± 0.18 vs. 0.70 ± 0.17, *p* < 0.001) compared to patients without DGBI symptom patterns (Table 3). Even though BMI and some metabolic parameters differed between patients with and without DGBI symptom patterns, these differences were minor and not considered clinically relevant.

**TABLE 3:** Comparison of baseline health outcomes in obesity with versus without comorbid symptoms compatible with a DGBI.

	Total cohort (*n* = 939)		
	With DGBI (*n* = 573)	Without DGBI (*n* = 366)	*p*
Gender (% female)	79.1 (75.5, 82.3)	67.5 (62.4, 72.3)	< 0.001
Gender (% male)	20.6 (17.4, 24.1)	32.5 (27.7, 37.6)	< 0.001
Class I obesity (%)	1.9 (1.0, 3.5)	2.5 (1.1, 4.7)	0.57
Class II obesity (%)	33.8 (29.9, 37.9)	28.5 (23.9, 33.5)	0.09
Class III obesity (%)	64.2 (60.1, 68.2)	69.0 (63.9, 73.7)	0.14
BMI (kg/m^2^)	41.65 ± 4.49	42.36 ± 5.25	0.03
HbA1c (mmol/mol)	39.48 ± 10.87	39.90 ± 11.30	0.59
Glucose (mmol/L)	6.54 ± 1.96	6.66 ± 2.05	0.43
LDL‐P cholesterol (nmol/L)	3.11 ± 0.84	2.99 ± 0.84	0.04
HDL‐P cholesterol (nmol/L)	1.20 ± 0.29	1.16 ± 0.27	0.04
Triglycerides (mmol/L)	1.81 ± 1.35	1.78 ± 1.15	0.71
Anxiety (BAI score)	10.0 [13.5]	5.0 [8.0]	< 0.001
Depression (PHQ‐9 score)	7.0 [9.0]	4.0 [6.0]	< 0.001
QoL (EQ5D index score)	0.64 ± 0.18	0.70 ± 0.17	< 0.001

Abbreviations: DGBI, disorder of gut‐brain interaction; HbA1c, Hemoglobin A1C; HDL‐P, high‐density lipoprotein particles; LDL‐P, low‐density lipoprotein particles; QoL, quality of life.

**FIGURE 2:**
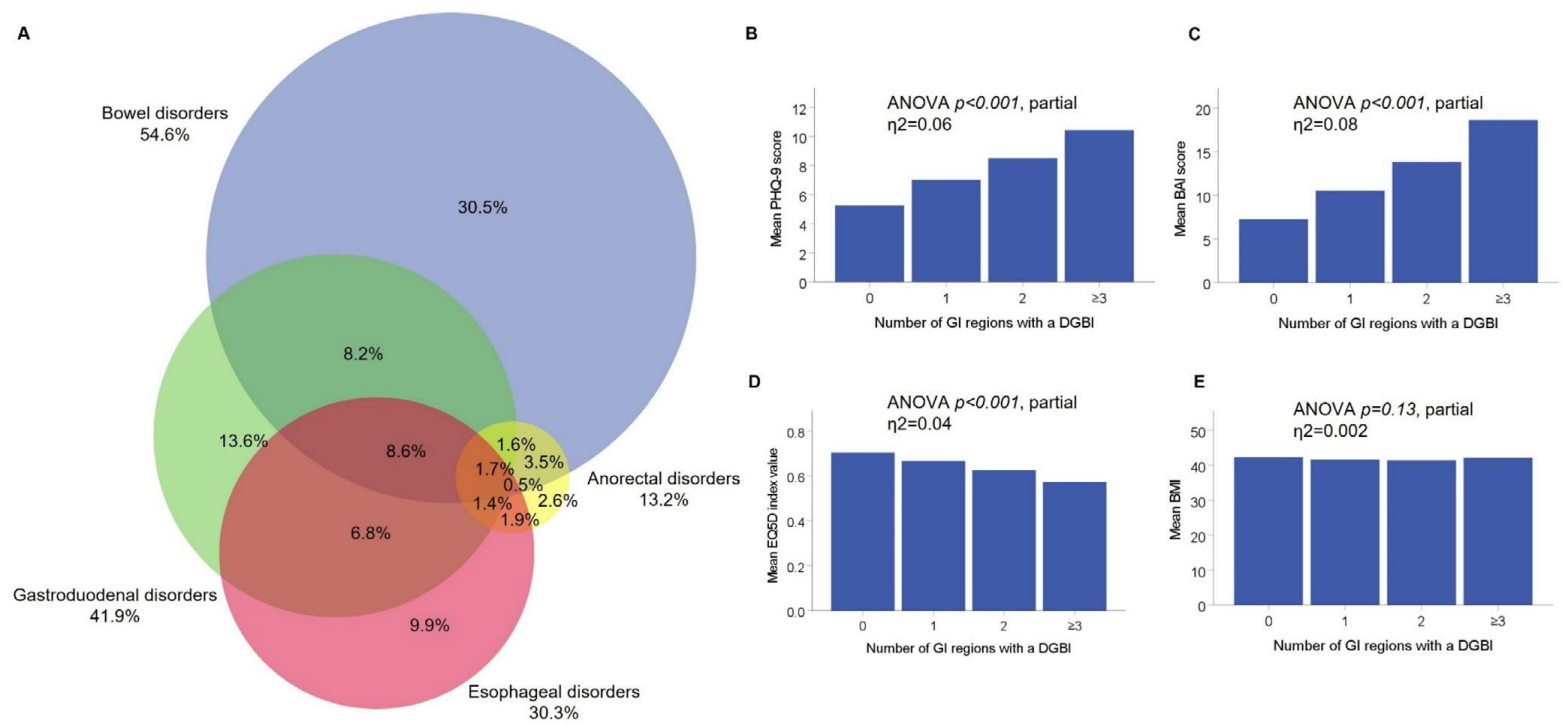
DGBI symptom patterns in multiple GI regions. (A) Venn diagram showing the overlap of DGBI regions within patients having at least one DGBI symptom pattern (*n* = 573). Not shown in figure; overlap between esophageal–bowel (7%), and gastroduodenal–anorectal (2%). The areas within the diagram are not exactly proportional to the numbers. (B–E) Association between the number of GI regions affected with DGBI and depression, anxiety, QoL, and BMI, respectively.

Of the 573 patients with DGBI symptom patterns, 57% had one, 30% had two, and 14% had three or four affected GI regions. In total, 44% of the patients had overlapping DGBI symptom patterns, with the most common two‐region overlap group being gastroduodenal–bowel (8%) (Figure 2A). With an increasing number of affected GI regions, there was a significant linear trend associated with more severe anxiety, depression, and poorer QoL with small to medium effect sizes, which was absent for BMI (Figure 2B–E).

### 3.4 Effect of Obesity Treatment on DGBI Symptom Patterns

To study the effect of obesity treatment on the prevalence of DGBI symptom patterns, we only analyzed the patients who completed the 2‐year follow‐up (*n* = 651) (mean BMI at follow‐up: 31.8 ± 6.1 kg/m^2^). Two years after obesity treatment, the proportion of patients with a comorbid DGBI symptom pattern decreased by 8% (Table 4). Overall, the proportion of patients having any esophageal disorder decreased substantially (9%), bowel disorders tended to decrease (4%), and the proportion of patients with any anorectal and gastroduodenal disorder tended to remain consistent after obesity treatment. Comparable outcomes were observed in both medical and RYGB treatments, with the exception of a substantial decrease in gastroduodenal disorders specifically in the medical and RYGB treatment groups, as opposed to the overall changes. After SG treatment, the prevalence of esophageal and bowel disorders remained stable, and gastroduodenal disorders substantially increased.

**TABLE 4:** Prevalence of DGBI diagnoses at baseline and follow‐up stratified by different treatments, only in patients who completed the 2‐year follow‐up (*n* = 651).

Diagnosis	Baseline				Follow‐up at 2 years			
	Overall (*n* = 651)	MT (*n* = 219)	RYGB (*n* = 279)	SG (*n* = 153)	Overall (*n* = 651)	MT (*n* = 219)	RYGB (*n* = 279)	SG (*n* = 153)
Esophageal disorders	23.7 (20.4, 27.1)	26.5 (20.8, 32.9)	24.7 (19.8, 30.2)	17.6 (12.0, 24.6)	14.9 (12.2, 18.0)	13.0 (8.7, 18.3)	14.7 (10.7, 19.4)	18.2 (12.4, 25.4)
Functional chest pain	6.1 (4.4, 8.2)	7.1 (4.0, 11.4)	5.4 (3.1, 8.8)	6.0 (2.8, 11.0)	4.3 (2.9, 6.2)	4.3 (2.0, 8.1)	3.7 (1.8, 6.7)	5.4 (2.4, 10.4)
Functional heartburn	13.7 (11.2, 16.6)	13.8 (9.5, 19.1)	17.6 (13.3, 22.6)	6.6 (3.2, 11.8)	6.5 (4.7, 8.8)	7.7 (4.5, 12.2)	3.0 (1.3, 5.7)	11.6 (6.9, 17.9)
Globus	2.6 (1.5, 4.2)	2.9 (1.1, 6.2)	2.0 (0.6, 4.5)	3.4 (1.1, 7.7)	0.7 (0.2, 1.7)	1.5 (0.3, 4.4)	0.4 (0.0, 2.1)	0.0 (0.0, 2.5)
Functional dysphagia	3.7 (2.4, 5.5)	5.5 (2.9, 9.5)	2.5 (1.0, 5.1)	3.3 (1.1, 7.5)	5.0 (3.4, 7.0)	1.4 (0.3, 4.2)	8.9 (5.8, 12.9)	2.8 (0.8, 6.9)
Gastroduodenal disorders	26.9 (23.5, 30.5)	26.5 (20.8, 32.9)	30.5 (25.1, 36.2)	20.9 (14.8, 28.2)	25.2 (21.9, 28.8)	19.4 (14.3, 25.4)	25.1 (20.1, 30.6)	33.8 (26.2, 42.0)
Functional dyspepsia	9.5 (7.3, 12.0)	14.2 (9.9, 19.6)	7.3 (4.5, 11.0)	6.6 (3.2, 11.8)	8.5 (6.5, 11.0)	6.3 (3.4, 10.6)	7.8 (4.9, 11.7)	13.0 (8.0, 19.6)
Belching disorder	7.9 (5.9, 10.2)	6.8 (3.9, 11.0)	9.0 (5.9, 13.0)	7.2 (3.7, 12.6)	7.9 (6.0, 10.3)	3.8 (1.7, 7.4)	7.7 (4.8, 11.5)	14.3 (9.1, 21.0)
Nausea and vomiting disorders	17.2 (14.4, 20.3)	14.6 (10.2, 20.0)	21.9 (17.2, 27.2)	12.4 (7.6, 18.7)	15.3 (12.6, 18.3)	13.3 (9.0, 18.6)	15.3 (11.2, 20.1)	18.2 (12.4, 25.4)
Rumination syndrome	0.3 (0.0, 1.1)	0.5 (0.0, 2.5)	0.4 (0.0, 2.0)	0.0 (0.0, 2.4)	0.8 (0.3, 1.8)	0.5 (0.0, 2.6)	0.4 (0.0, 2.0)	2.0 (0.4, 5.8)
Bowel disorders	37.6 (33.9, 41.5)	38.4 (31.9, 45.1)	36.2 (30.6, 42.1)	39.2 (31.4, 47.4)	33.5 (29.9, 37.3)	31.7 (25.5, 38.3)	31.5 (26.1, 37.3)	39.9 (32.1, 48.1)
Irritable bowel syndrome	20.2 (17.2, 23.5)	25.7 (20.0, 32.0)	15.5 (11.4, 20.3)	21.1 (14.9, 28.4)	14.2 (11.6, 17.2)	13.3 (9.1, 18.5)	14.4 (10.5, 19.1)	15.1 (8.9, 21.8)
IBS‐C	3.3 (2.1, 5.0)	4.3 (2.0, 8.0)	2.6 (1.0, 5.2)	3.4 (1.1, 7.8)	4.2 (2.8, 6.1)	3.0 (1.1, 6.4)	3.7 (1.8, 6.7)	6.9 (3.4, 12.3)
IBS‐D	5.9 (4.2, 8.0)	8.6 (5.2, 13.2)	5.1 (2.8, 8.4)	3.4 (1.1, 7.8)	2.9 (1.7, 4.6)	2.5 (0.8, 5.7)	4.0 (2.0, 7.1)	1.4 (0.2, 4.9)
IBS‐M	9.8 (7.6, 12.4)	12.4 (8.2, 17.6)	7.3 (4.5, 11.0)	10.9 (6.4, 17.1)	6.0 (4.3, 8.2)	7.0 (3.9, 11.4)	6.3 (3.7, 9.8)	4.1 (1.5, 8.8)
IBS‐U	1.0 (0.3, 2.1)	1.0 (0.1, 3.4)	0.4 (0.0, 2.0)	2.0 (0.4, 5.8)	1.5 (0.7, 2.7)	1.0 (0.1, 3.5)	0.7 (0.1, 2.6)	3.4 (1.1, 7.9)
Functional constipation	11.1 (8.8, 13.7)	9.1 (5.7, 13.8)	12.2 (8.6, 16.6)	11.8 (7.1, 18.0)	16.8 (14.0, 19.9)	13.3 (9.1, 18.5)	15.1 (11.1, 19.8)	24.8 (18.2, 32.5)
Functional diarrhea	4.3 (2.9, 6.2)	2.3 (0.7, 5.3)	5.4 (3.1, 8.7)	5.3 (2.3, 10.1)	0.0 (0.0, 0.6)	0.0 (0.0, 1.7)	0.0 (0.0, 1.3)	0.0 (0.0, 2.4)
Functional abdominal bloating/distention	5.4 (3.4, 8.3)	3.9 (1.3, 8.9)	7.1 (3.6, 12.3)	4.8 (1.3, 11.7)	3.3 (1.9, 5.2)	6.8 (3.5, 11.9)	2.6 (1.0, 5.5)	0.0 (0.0, 3.0)
Anorectal disorders	8.2 (6.2, 10.6)	12.4 (8.3, 17.5)	6.8 (4.1, 10.4)	4.6 (1.9, 9.3)	8.4 (6.4, 10.8)	10.5 (6.7, 15.5)	8.4 (5.4, 12.3)	5.4 (2.4, 10.4)
Fecal incontinence	8.1 (6.1, 10.5)	12.0 (8.0, 17.1)	6.8 (4.2, 10.5)	4.7 (1.9, 9.4)	7.0 (5.1, 9.3)	8.2 (4.8, 12.8)	7.3 (4.5, 11.0)	4.8 (2.0, 9.7)
Functional anorectal pain	0.3 (0.0, 1.1)	0.9 (0.1, 3.3)	0.0 (0.0, 1.3)	0.0 (0.0, 2.4)	1.8 (0.9, 3.1)	3.4 (1.4, 6.8)	1.1 (0.2, 3.2)	0.7 (0.0, 3.8)

*Note:* All patients who were not appointed to a treatment group (*n* = 55) or who were lost to follow‐up (*n* = 288) were not considered in this analysis.

Abbreviations: IBS‐C, irritable bowel syndrome with predominant constipation; IBS‐D, irritable bowel syndrome with predominant diarrhea; IBS‐M, irritable bowel syndrome with mixed bowel habits; IBS‐U, irritable bowel syndrome unsubtyped; MT, medical treatment; RYGB, Roux‐en‐Y gastric bypass; SG, sleeve gastrectomy.

**FIGURE 3:**
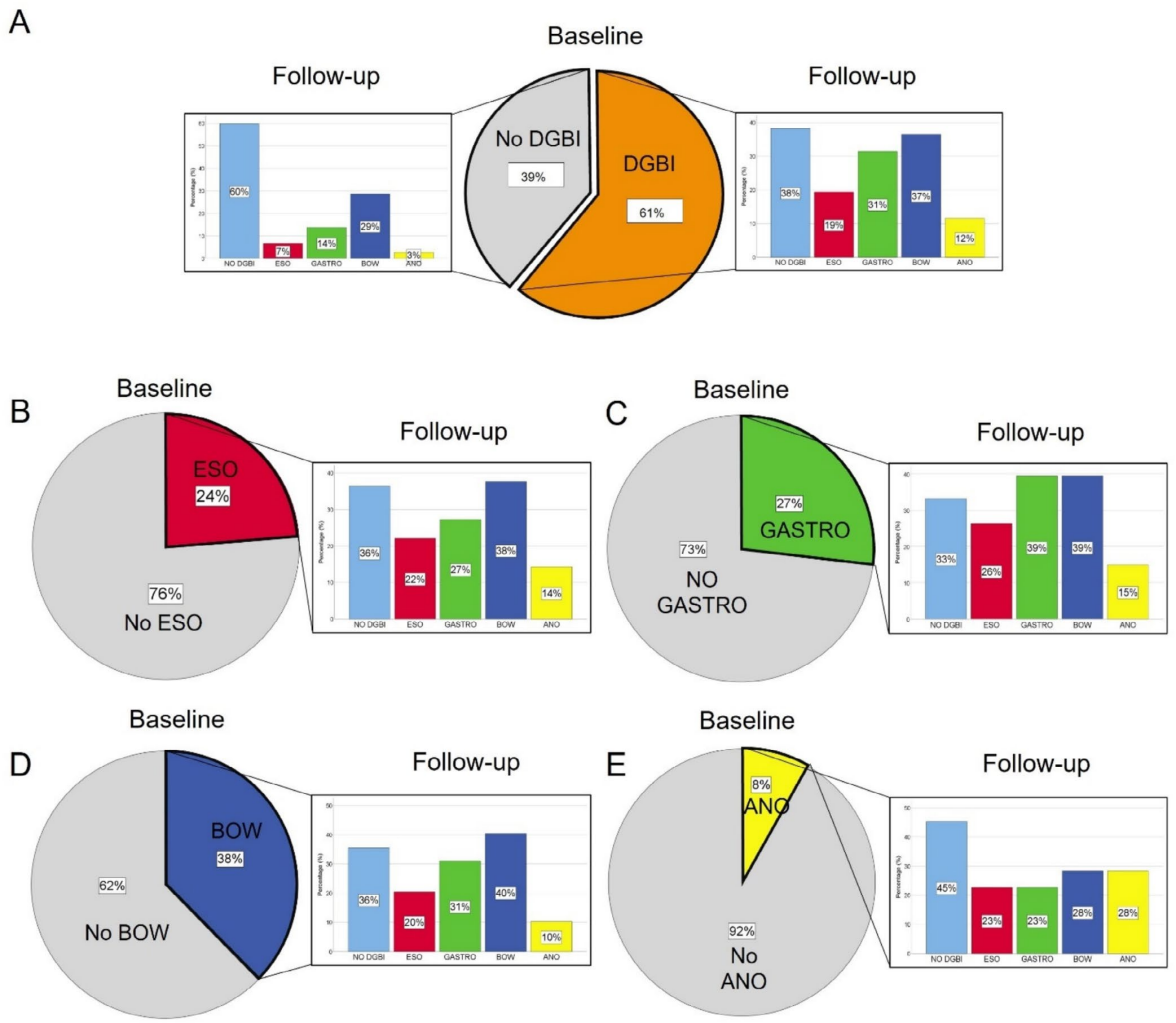
DGBI symptom patterns at baseline and follow‐up. Due to the presence of overlapping conditions, the sum of the separate proportions in the follow‐up conditions could be more than 100%. (A) At least one DGBI symptom pattern at baseline. (B–E) Symptom pattern compatible with an esophageal, gastroduodenal, bowel, and anorectal DGBI at baseline, respectively.

There was a substantial shift between baseline and follow‐up DGBI symptom patterns across all GI regions (Figure 3). Of the patients who initially showed DGBI symptoms, 38% no longer had these symptoms after undergoing obesity treatment. Conversely, 40% of the patients who did not show DGBI symptoms at the start developed new DGBI symptoms by the follow‐up. Figures [Link], [Link], [Link] display results in the medical, RYGB, and SG treatment arms. Consistent findings were noted in the medical and RYGB arms. In SG, the proportion of patients with a DGBI symptom profile at baseline not reporting it at follow‐up was substantially lower (29%).

In the subgroup of diabetes patients (Tables [Link], [Link], [Link], [Link], [Link]), there were no significant differences in results, except for a slightly higher proportion of men, class II obesity, older patients, and an increased occurrence of fecal incontinence. Detailed minor differences are provided in the table notes.

## 4 Discussion

This study investigated the prevalence of DGBI symptom patterns in obesity, associated factors of DGBI in obesity, and obesity treatment effect. DGBI symptom patterns across all anatomical GI regions were common in obesity, and having a comorbid DGBI symptom profile was associated with more severe psychological distress, poorer QoL, but not with BMI. Overall, the prevalence of DGBI symptom patterns slightly decreased after obesity treatment. However, we observed a substantial shift between baseline and follow‐up symptom patterns, indicating that different obesity treatments could potentially have both an attenuating and amplifying impact on DGBI symptom profiles in obesity.

The global prevalence of DGBI in the general population is estimated to be 40% [26]. While this highlights the commonality of DGBI, it also emphasizes that the 61% prevalence of DGBI symptom patterns observed in patients with obesity is notably higher. However, it is important to consider that comparing the prevalence in obesity treatment populations to the general population may introduce bias. Individuals seeking obesity treatment are more likely to interact with the healthcare system, potentially increasing the likelihood of DGBI diagnosis or symptom reporting. Furthermore, the comparison is between DGBI symptom patterns, not formal diagnoses, in our cohort, while the general population data often stems from epidemiological studies. A similar pattern holds for specific DGBI, such as IBS, where the prevalence of Rome III IBS is estimated to be 9% in the general population, compared to the 21% found in our cohort of patients with obesity [27]. The link between IBS and obesity has been inconsistent in prior studies, with associations being positive [6, 28], insignificant when adjusted for confounders [10, 11, 12, 13], positive in a certain subgroup [7, 29, 30] or even negative [8, 9]. Nonetheless, our findings suggest a high prevalence of IBS symptom patterns in patients with obesity. Other commonly reported symptom patterns in our study population were symptoms of nausea and vomiting disorders and functional heartburn. Nausea and vomiting symptoms have been more consistently associated with obesity [8]. Nevertheless, in the majority of studies, only vomiting emerged as the factor that correlates most persistently with obesity [3, 6, 10]. Functional heartburn is a disorder included in the GERD spectrum. To formally classify patients across the GERD spectrum, upper GI endoscopy and 24‐h pH recordings are needed, which were not part of this study protocol. Numerous studies have shown that obesity or an increased BMI is a risk factor for the development of GERD or GERD‐related symptoms [3, 10, 28, 31, 32, 33, 34]. Hence, DGBI symptom patterns across the complete GI tract with potential overlapping symptom patterns are highly prevalent in patients with obesity. Overall, symptoms linked to bowel disorders were the most common in our study, mainly due to the contribution of IBS symptoms, followed by symptom patterns of gastroduodenal and esophageal disorders, and the less prevalent anorectal disorders.

Comorbid DGBI symptoms in obesity were associated with poorer health outcomes, including more severe psychological distress, poorer QoL, but not with BMI. This finding is not surprising since it has been shown that there is an inverse relationship between multimorbidity or comorbidity and QoL [35]. The same pattern has been observed in the DGBI literature, where having DGBI in multiple anatomic GI regions is positively associated with symptom severity and health care utilization [36]. The absence of a clinically relevant relationship between BMI and comorbid DGBI symptom patterns has also been observed in patients with obesity who have comorbid GERD symptoms [37]. Given the observed associations between DGBI symptom patterns, quality of life, anxiety, and depression, addressing DGBI symptoms according to established clinical guidelines is essential [38, 39]. Physicians should therefore recognize DGBI symptoms as potential comorbidities in patients with obesity, as incorporating this aspect could be valuable in managing this subgroup effectively.

We assessed the effect of obesity treatment on DGBI symptom prevalence. When all treatment groups were pooled, the overall prevalence of symptoms of esophageal disorders decreased substantially, and symptoms of gastroduodenal disorders stagnated. However, in patients receiving SG, the prevalence of esophageal and gastroduodenal disorders remained fairly consistent or substantially increased, that is, almost doubled, for specific disorders such as functional heartburn. Research has also shown that the use of acid‐reducing medication and symptoms of heartburn and regurgitation are increased in patients who were treated with SG [40]. This is in line with preexisting knowledge of the physiologic effects of SG surgery. Additionally, clinical guidelines recommend that GERD is a contraindication for SG, and acid‐reducing medication should be prescribed after SG. Our findings align with this perspective, underscoring the importance of customizing obesity treatment to address any GI‐related symptoms or diagnoses identified prior to the intervention. In patients treated with RYGB, the only esophageal symptom pattern becoming more prevalent after surgery was functional dysphagia. Similar observations have been made in a previous study that indicated that dysphagia scores are equivalent to control subjects preoperatively but increase significantly in obesity after RYGB [41]. This consideration should be integrated into the decision‐making process for selecting the appropriate obesity treatment, especially in cases where dysphagia symptoms are already evident prior to the intervention. Overall, symptoms of bowel disorders decreased by 6% in our cohort. Dietary alterations, which occurred in the medical treatment arm, but likely also in the patients receiving surgery, might have an important effect on bowel habit, including diarrhea and constipation. Switching from a high‐fat diet to a low‐fat diet affects bile acid release, potentially resulting in a decreased production of bile, a natural laxative. This specific shift, along with all changes in DGBI symptom profiles observed in this study, may play a role in the decrease of IBS‐D and functional diarrhea symptoms. Other factors, such as lifestyle changes that often come with dietary adjustments and potential alterations in gut microbiota after surgical interventions, could also contribute to these changes. However, constipation symptom patterns seem to increase in all treatment arms, with the most pronounced effect in the SG arm. Postoperative constipation after bariatric surgery is a well‐described early postoperative phenomenon that stems from altering the anatomy of the GI tract and therefore inducing changes in patients' bowel habits [42]. In addition, constipation in obesity has been shown to be associated with polypharmacy, which might partly explain the increase [43]. However, here, bowel symptoms may also be linked to organic disorders, such as cholelithiasis and ulcers, which can develop following obesity treatments, particularly RYGB, as noted in a previous study [44]. Hence, these findings are important for treatment decisions and follow‐up of patients with obesity. Our findings support previous literature showing that SG should not be the surgical technique for obese patients with GERD symptoms and potentially also other comorbid DGBI symptom patterns. It remains uncertain whether the surgical technique, dietary and lifestyle alteration, or weight loss resulting from the interventions is the driving force behind the improvement in symptoms. Hence, future research should focus on unraveling the mechanisms through which GI symptoms occur in obesity before and after treatment.

Lastly, we briefly note differences in analyses between the complete cohort and the diabetes subgroup. Demographic variations align with literature indicating higher diabetes prevalence in men and older individuals [45, 46]. Neurological disorders secondary to diabetes are consistently linked to motor function abnormalities, potentially explaining the increased fecal incontinence prevalence [47].

Strengths of our study include the large sample size, enhancing statistical power and generalizability. Most studies start with a general population sample of which they select either a subgroup with obesity or DGBI, which often leads to a smaller sample size. Secondly, we defined DGBI symptom patterns across the entire GI tract instead of focusing on one specific DGBI, such as IBS. Thirdly, we identified DGBI symptom patterns before and after obesity treatment, allowing us to study both the baseline prevalence and the effect of three different obesity treatments. However, a substantial proportion of the patients were lost in follow‐up after 2 years, which influences our results. We used the validated Rome III questionnaire to identify DGBI symptom patterns. Our strength in using the Rome III questionnaire is also a limitation of our study, since we did not use the more up‐to‐date Rome IV questionnaire. However, this version of the survey was not published at the time when the study started. In addition, DGBI symptom patterns were not confirmed by a physician. Therefore, we cannot rule out medication use or more uncommon structural diseases as potential sources of the DGBI symptom patterns we identified. Due to the study design not allowing for the inclusion of a non‐obese control group, our ability to investigate potential causal relationships between DGBI symptom patterns, obesity, and its treatment is limited. Our study focused on a cohort of patients with obesity referred for treatment, which may introduce selection bias and restrict the generalizability of the findings. Additionally, DGBI symptoms can fluctuate over time, even without intervention, making it more difficult to interpret symptom changes without a non‐obese control group.

In conclusion, this study demonstrates that a substantial proportion of patients with obesity experience DGBI symptom patterns across all anatomical GI regions, with potential overlap between conditions. DGBI symptom patterns in obesity are not associated with the degree of obesity, but with poorer QoL and more severe psychological distress. Obesity treatment is associated with an overall decrease in the prevalence of DGBI. However, the prevalence of some specific DGBI symptom patterns increased. There is a substantial shift between baseline and follow‐up symptom patterns, with notable differences between obesity treatment options.

## Author Contributions

E.C., J.H., M.S. planned and initiated this particular analysis. E.C., M.B. conducted the analyses and interpreted the data before E.C. drafted the manuscript. G.H., K.M., M.E., B.E., V.W., L.F. designed the original BASUN study and collected the data. All authors reviewed the manuscripts. J.T., H.T., M.S. supervised the overall project.

## Conflicts of Interest

G.H. reports personal fees from AstraZeneca and NovoNordisk. B.E. reports personal fees from Amgen, AstraZeneca, Boehringer Ingelheim, Eli Lilly, Merck Sharp and Dohme, Mundipharma, NovoNordisk, RLS Global, and Sanofi. J.T. has given scientific advice to Adare, AlfaWassermann, Allergan, Arena, Bayer, Christian Hansen, Clasado, Danone, Devintec, Falk, Grünenthal, Ironwood, Janssen, Kiowa Kirin, Menarini, Mylan, Neurogastrx, Neutec, Novartis, Noventure, Nutricia, Shionogi, Shire, Takeda, Theravance, Tramedico, Truvion, Tsumura, Zealand, and Zeria Pharmaceutical; received research support from Shire, Sofar, and Tsumura; and served on the Speakers Bureau for Abbott, Allergan, AstraZeneca, Janssen, Kyowa Kirin, Menarini, Mylan, Novartis, Shire, Takeda, Truvion, and Zeria Pharmaceutical. H.T. has received personal fees from Galapagos, Tillotts Pharma, Shire, Takeda, Cinclus Pharma, Dr. Falk Pharma Gmbh, and Vipun Medical. M.S. has received grants and personal fees from BioGaia and Danone Nutricia Research; personal fees from Ironwood, Biocodex, Tillotts, Kyowa Kirin, Takeda, Abbvie, Cinclus Pharma, Renapharma, Sanofi, Janssen Immunology, Pfizer, Mayoly, and Bromatech; and grants from Genetic Analysis AS. All reported COIs were outside the submitted work. Other authors declare no conflicts of interest.

## Supporting information


**Figure S1.** Proportion of patients with a DGBI symptom profile at baseline and with or without a symptom pattern at follow‐up in the medical treatment arm. The sum of the separate proportions in the follow‐up conditions can be more than 100% since patients were able to have overlapping conditions. (A) At least one symptom pattern compatible with a DGBI at baseline, (B) symptom pattern compatible with an esophageal DGBI at baseline, (C) symptom pattern compatible with a gastroduodenal DGBI at baseline, (D) symptom pattern compatible with a bowel DGBI at baseline, (E) symptom pattern compatible with an anorectal DGBI at baseline.

**Figure S2.** Proportion of patients with a DGBI symptom profile at baseline and with or without a symptom pattern at follow‐up in the RYGB treatment arm. The sum of the separate proportions in the follow‐up conditions can be more than 100% since patients were able to have overlapping conditions. (A) At least one symptom pattern compatible with a DGBI at baseline, (B) symptom pattern compatible with an esophageal DGBI at baseline, (C) symptom pattern compatible with a gastroduodenal DGBI at baseline, (D) symptom pattern compatible with a bowel DGBI at baseline, (E) symptom pattern compatible with an anorectal DGBI at baseline.

**Figure S3.** Proportion of patients with a DGBI symptom profile at baseline and with or without a symptom pattern at follow‐up in the SG treatment arm. The sum of the separate proportions in the follow‐up conditions can be more than 100% since patients were able to have overlapping conditions. (A) At least one symptom pattern compatible with a DGBI at baseline, (B) symptom pattern compatible with an esophageal DGBI at baseline, (C) symptom pattern compatible with a gastroduodenal DGBI at baseline, (D) symptom pattern compatible with a bowel DGBI at baseline, (E) symptom pattern compatible with an anorectal DGBI at baseline.

Table S1.

Table S2.

Table S3.

Table S4.

Table S5.

## Data Availability

The data that support the findings of this study are available on request from the corresponding author. The data are not publicly available due to privacy or ethical restrictions.
