# Supplementary figures and images for: Prevalence and Factors Associated With Symptom Profiles of Disorders of Gut‐Brain Interaction in Obesity Before and After Treatment
Source: Neurogastroenterol Motil. 2025 Mar 10;38:e70017. doi: 10.1111/nmo.70017 (PMC13121869; doi:10.1111/nmo.70017)

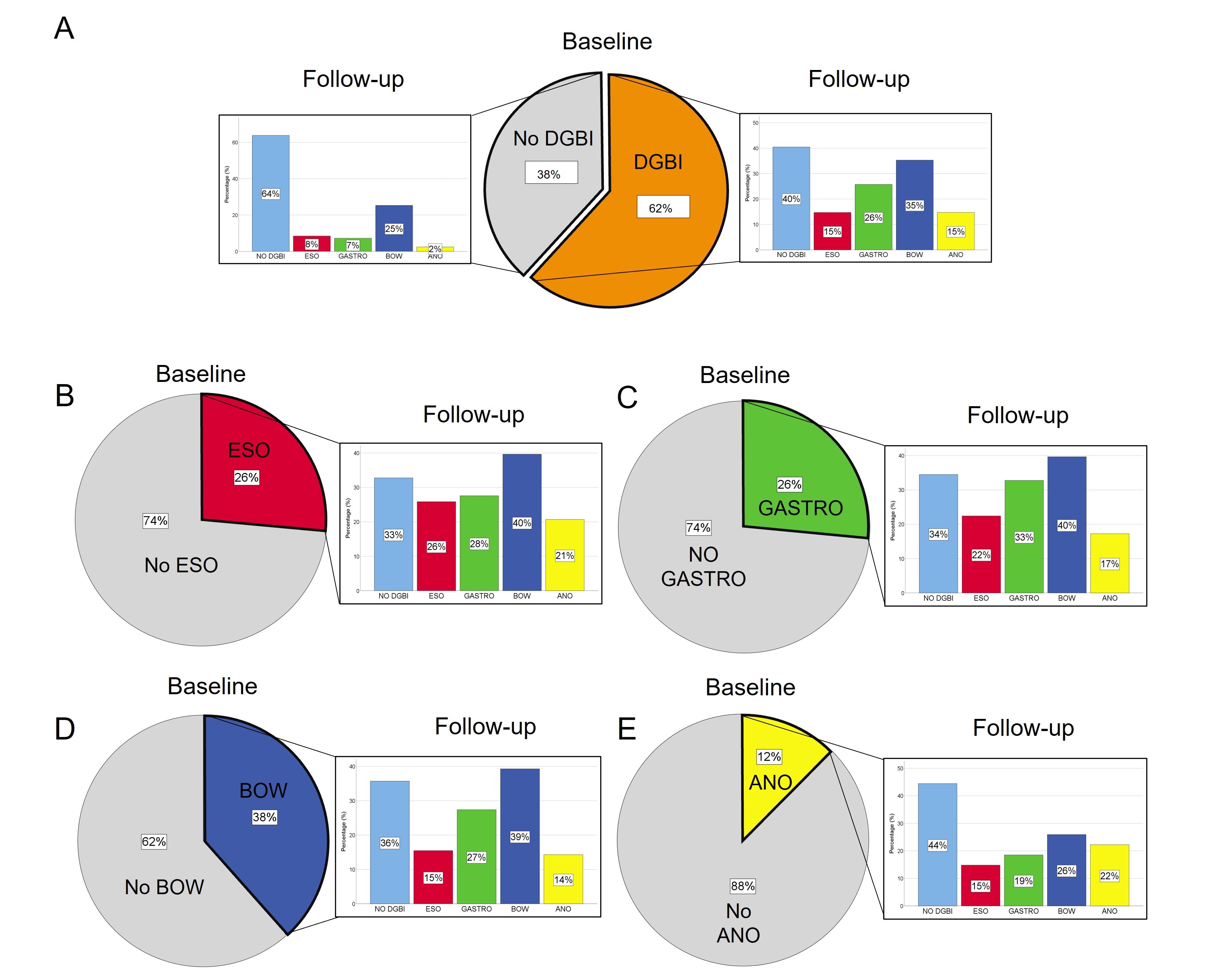

Supplement: Supplementary file 1 — Figure S1. Proportion of patients with a DGBI symptom profile at baseline and with or without a symptom pattern at follow‐up in the medical treatment arm. The sum of the separate proportions in the follow‐up conditions can be more than 100% since patients were able to have overlapping conditions. (A) At least one symptom pattern compatible with a DGBI at baseline, (B) symptom pattern compatible with an esophageal DGBI at baseline, (C) symptom pattern compatible with a gastroduodenal DGBI at baseline, (D) symptom pattern compatible with a bowel DGBI at baseline, (E) symptom pattern compatible with an anorectal DGBI at baseline. [file NMO-38-e70017-s001.jpg]

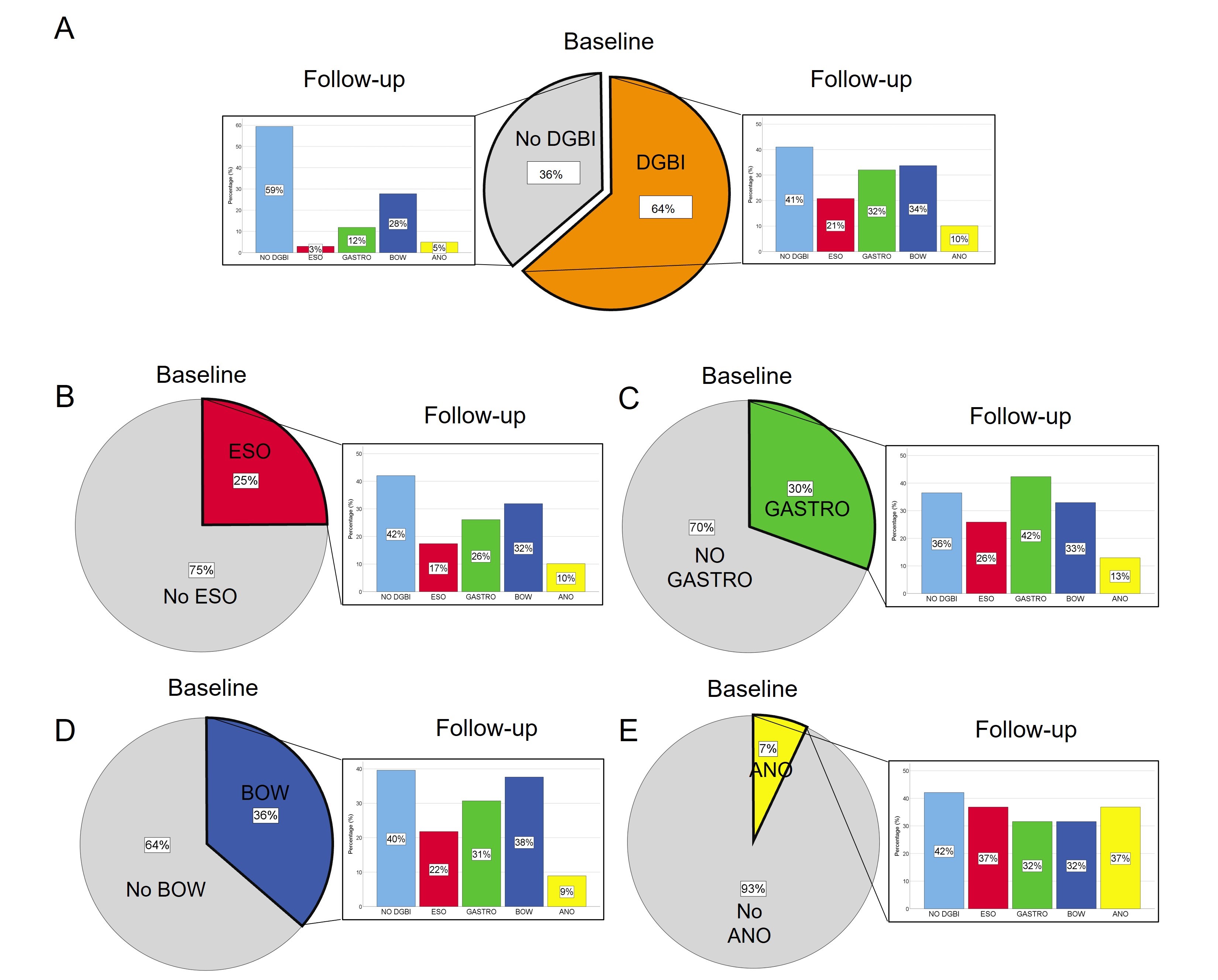

Supplement: Supplementary file 2 — Figure S2. Proportion of patients with a DGBI symptom profile at baseline and with or without a symptom pattern at follow‐up in the RYGB treatment arm. The sum of the separate proportions in the follow‐up conditions can be more than 100% since patients were able to have overlapping conditions. (A) At least one symptom pattern compatible with a DGBI at baseline, (B) symptom pattern compatible with an esophageal DGBI at baseline, (C) symptom pattern compatible with a gastroduodenal DGBI at baseline, (D) symptom pattern compatible with a bowel DGBI at baseline, (E) symptom pattern compatible with an anorectal DGBI at baseline. [file NMO-38-e70017-s003.jpg]

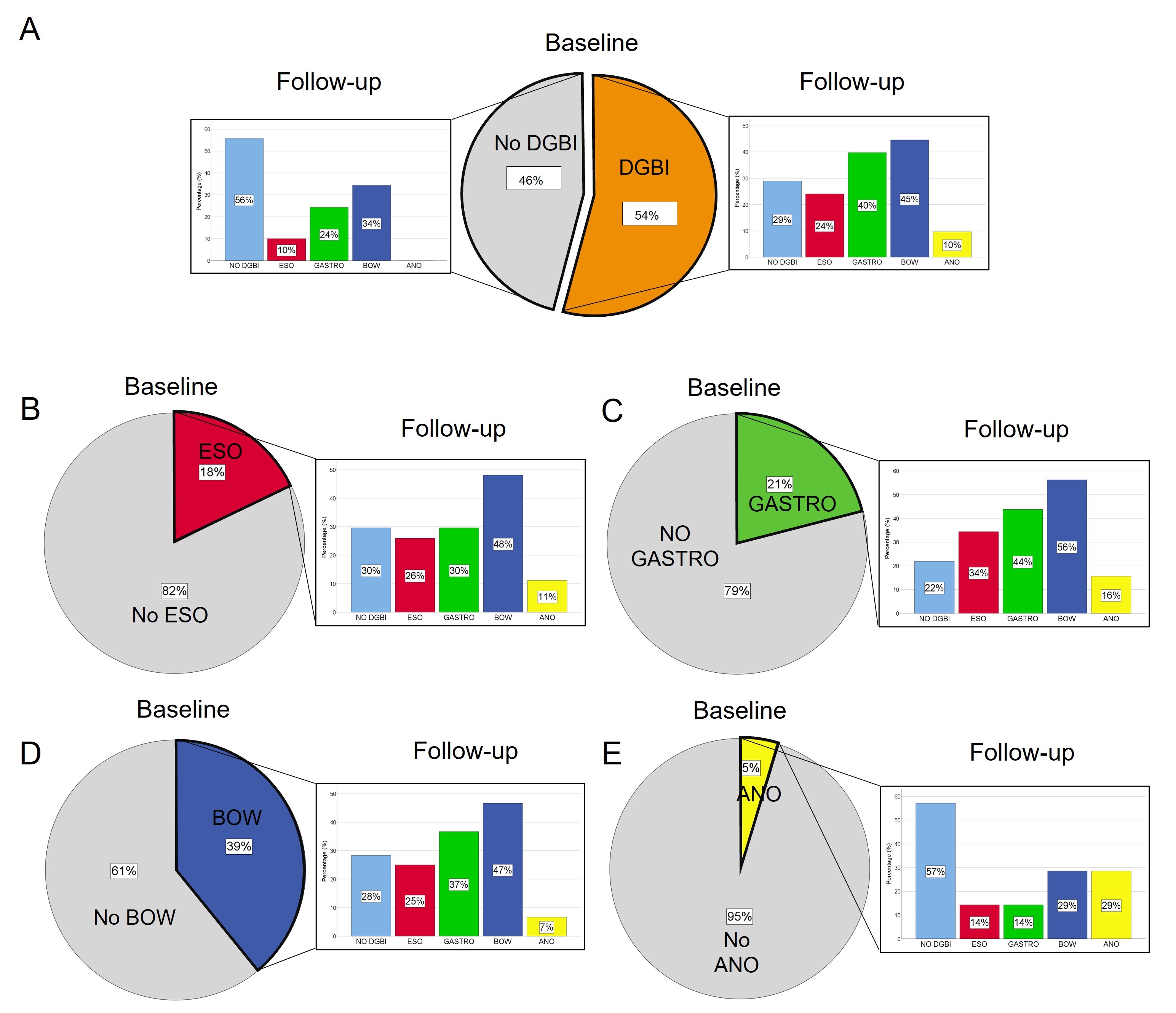

Supplement: Supplementary file 3 — Figure S3. Proportion of patients with a DGBI symptom profile at baseline and with or without a symptom pattern at follow‐up in the SG treatment arm. The sum of the separate proportions in the follow‐up conditions can be more than 100% since patients were able to have overlapping conditions. (A) At least one symptom pattern compatible with a DGBI at baseline, (B) symptom pattern compatible with an esophageal DGBI at baseline, (C) symptom pattern compatible with a gastroduodenal DGBI at baseline, (D) symptom pattern compatible with a bowel DGBI at baseline, (E) symptom pattern compatible with an anorectal DGBI at baseline. [file NMO-38-e70017-s005.jpg]
