# Supplementary material for: Prevalence and Factors Associated With Symptom Profiles of Disorders of Gut‐Brain Interaction in Obesity Before and After Treatment
Source: Neurogastroenterol Motil. 2025 Mar 10;38:e70017. doi: 10.1111/nmo.70017 (PMC13121869; doi:10.1111/nmo.70017)
Supplement: Supplementary file 4 — Table S1. [file NMO-38-e70017-s004.docx]

**Supplementary table 1.** Demographic factors of patients with comorbid diabetes stratified by different treatments at baseline

| ***Demographic (%)*** | **Baseline cohort stratified** | | | | |
| --- | --- | --- | --- | --- | --- |
|  | **Overall**  **(n=144)** | **MT (n=44)** | **RYGB (n=56)** | **SG (n=24)** | **Discontinued (n=20)** |
| *Gender* |  |  |  |  |  |
| Female | 63.2 (54.8, 71.1) | 59.1 (43.2, 73.7) | 66.1 (52.2, 78.2) | 66.7 (44.7, 84.4) | 60.0 (36.1, 80.9) |
| Male | 36.8 (28.9, 45.2) | 40.9 (26.3, 56.8) | 33.9 (21.8, 47.8) | 33.3 (15.6, 55.3) | 40.0 (19.1, 63.9) |
| Other | 0.0 (0.0, 2.5) | 0.0 (0.0, 8.0) | 0.0 (0.0, 6.4) | 0.0 (0.0, 14.2) | 0.0 (0.0, 16.8) |
| *Age groups (years)* |  |  |  |  |  |
| 18-29 | 4.9 (2.0), 9.8) | 2.3 (0.1, 12.0) | 5.4 (1.1, 14.9) | 4.2 (0.1, 21.1) | 10.5 (1.3, 33.1) |
| 30-44 | 22.4 (15.8, 30.1) | 4.5 (0.6, 15.5) | 23.2 (13.0, 36.4) | 41.7 (22.1, 63.4) | 36.8 (16.3, 61.6) |
| 45-59 | 52.4 (43.9, 60.9) | 45.5 (30.4, 61.2) | 62.5 (48.5, 75.1) | 54.2 (32.8, 74.4) | 36.8 (16.3, 61.6) |
| 60-74 | 18.9 (12.8, 26.3) | 43.2 (28.3, 59.0) | 8.9 (3.0, 19.6) | 0.0 (0.0, 14.2) | 15.8 (3.4, 39.6) |
| ≥75 | 1.4 (0.2, 5.0) | 4.5 (0.6, 15.5) | 0.0 (0.0, 6.4) | 0.0 (0.0, 14.2) | 0.0 (0.0, 17.6) |
| *BMI groups (kg/m²)* |  |  |  |  |  |
| Obese I^a^ | 4.3 (1.6, 9.2) | 6.8 (1.4, 18.7) | 3.6 (0.4, 12.3) | 4.2 (0.1, 21.1) | 0.0 (0.0, 23.2) |
| Obese II^b^ | 48.6 (40.0, 57.2) | 43.2 (28.3, 59.0) | 50.0 (36.3, 63.7) | 41.7 (22.1, 63.4) | 71.4 (41.9, 91.6) |
| Obese III ^c^ | 47.1 (38.6, 55.8) | 50.0 (34.6, 65.4) | 46.4 (33.3, 60.3) | 54.2 (32.8, 74.4) | 28.6 (8.4, 58.1) |
| *Social status* |  |  |  |  |  |
| Married | 45.1 (36.8, 53.6) | 40.9 (26.3, 56.8) | 48.2 (34.7, 62.0) | 45.8 (25.6, 67.2) | 45.0 (23.1, 68.5) |
| Lives with parent(s) | 0.0 (0.0, 2.5) | 0.0 (0.0, 8.0) | 0.0 (0.0, 6.4) | 0.0 (0.0, 14.2) | 0.0 (0.0, 16.8) |
| Cohabiting | 19.4 (13.3, 26.9) | 15.9 (6.6, 30.1) | 26.8 (15.8, 40.3) | 12.5 (2.7, 32.4) | 15.0 (3.2, 37.9) |
| Single | 31.3 (23.8, 39.5) | 36.4 (22.4, 52.2) | 23.2 (13.0, 36.4) | 41.7 (22.1, 63.4) | 30.0 (11.9, 54.3) |
| *Education* |  |  |  |  |  |
| Primary | 15.2 (9.7, 22.3) | 30.0 (16.6, 46.5) | 9.3 (3.1, 20.3) | 8.3 (1.0, 27.0) | 10.0 (1.2, 31.7) |
| Secondary | 53.6 (44.9, 62.1) | 45.0 (29.3, 61.5) | 61.1 (46.9, 74.1) | 50.0 (29.1, 70.9) | 55.0 (31.5, 76.9) |
| Tertiary | 31.2 (23.6, 39.6) | 25.0 (12.7, 41.2) | 29.6 (18.0, 43.6) | 41.7 (22.1, 63.4) | 35.0 (15.4, 59.2) |

NOTE: The table displays column percentages. MT: medical treatment, RYGB: Roux-en-Y gastric bypass, SG: sleeve gastrectomy, a: 30 kg/m^2^ < BMI < 34.9 kg/m^2^, b: 35 kg/m^2^ < BMI < 39.9 kg/m^2^, c: BMI >40 kg/m^2^.
