# Supplementary material for: Prevalence and Factors Associated With Symptom Profiles of Disorders of Gut‐Brain Interaction in Obesity Before and After Treatment
Source: Neurogastroenterol Motil. 2025 Mar 10;38:e70017. doi: 10.1111/nmo.70017 (PMC13121869; doi:10.1111/nmo.70017)
Supplement: Supplementary file 5 — Table S2. [file NMO-38-e70017-s006.docx]

**Supplementary table 2.** Prevalence of DGBI diagnoses at baseline and follow-up stratified by different treatments, diabetes only (n=144)

| ***Diagnosis*** | **Overall (n=144)** | **MT (n=44)** | **RYGB (n=56)** | **SG (n=24)** | **Discontinued (n=20)** |  |
| --- | --- | --- | --- | --- | --- | --- |
| *Esophageal disorders* | 20.1 (13.9, 27.6) | 25.0 (13.2, 40.3) | 17.9 (8.9, 30.4) | 16.7 (4.7, 37.4) | 20.0 (5.7, 43.7) | |
| Functional chest pain | 4.3 (1.6, 9.0) | 9.5 (2.7, 22.6) | 1.8 (0.0, 9.7) | 0.0 (0.0, 14.2) | 5.0 (0.1, 24.9) | |
| Functional heartburn | 11.1 (6.5, 17.4) | 11.4 (3.8, 24.6) | 10.7 (4.0, 21.9) | 8.3 (1.0, 27.0) | 15.0 (3.2, 37.9) | |
| Globus | 2.3 (0.5, 6.5) | 0.0 (0.0, 8.6) | 2.0 (0.1, 10.9) | 8.7 (1.1, 28.0) | 0.0 (0.0, 17.6) | |
| Functional dysphagia | 4.9 (2.0, 9.8) | 6.8 (1.4, 18.7) | 5.5 (1.1, 15.1) | 4.2 (0.1, 21.1) | 0.0 (0.0, 16.8) | |
| *Gastroduodenal disorders* | 20.8 (14.5, 28.4) | 29.5 (16.8, 45.2) | 16.1 (7.6, 28.3) | 20.8 (7.1, 42.2) | 15.0 (3.2, 37.9) | |
| Functional dyspepsia | 7.9 (4.0, 13.6) | 19.0 (8.6, 34.1) | 1.9 (0.0, 10.1) | 4.2 (0.1, 21.1) | 5.0 (0.1, 24.9) | |
| Belching disorder | 7.9 (4.0, 13.6) | 9.1 (2.5, 31.7) | 5.8 (1.2, 15.9) | 8.3 (1.0, 27.0) | 10.0 (1.2, 31.7) | |
| Nausea and vomiting disorders | 12.5 (7.6, 19.0) | 15.9 (6.6, 30.1) | 12.5 (5.2, 24.1) | 8.3 (1.0, 27.0) | 10.0 (1.2, 31.7) | |
| Rumination syndrome | 0.0 (.0, 2.5) | 0.0 (0.0, 8.0) | 0.0 (0.0, 6.5) | 0.0 (0.0, 14.2) | 0.0 (0.0, 16.8) | |
| *Bowel disorders* | 39.2 (31.1, 47.7) | 27.3 (15.0, 42.8) | 40.0 (27.0, 54.1) | 45.8 (25.6, 67.2) | 55.0 (31.5, 76.9) | |
| Irritable bowel syndrome | 22.0 (15.5, 29.7) | 18.2 (8.2, 32.7) | 18.5 (9.3, 31.4) | 26.1 (10.2, 48.4) | 35.0 (15.4, 59.2) | |
| IBS-C | 2.2 (0.4, 6.2) | 0.0 (0.0, 8.0) | 1.9 (0.0, 9.9) | 4.8 (0.1, 23.8) | 5.0 (0.1, 24.9) | |
| IBS-D | 7.9 (4.0, 13.7) | 9.1 (2.5, 21.7) | 7.4 (2.1, 17.9) | 9.5 (1.2, 30.4) | 5.0 (0.1, 24.9) | |
| IBS-M | 10.8 (6.2, 17.2) | 6.8 (1.4, 18.7) | 9.3 (3.1, 20.3) | 9.5 (1.2, 30.4) | 25.0 (8.7, 49.1) | |
| IBS-U | 0.7 (0.0, 3.9) | 2.3 (0.1, 12.0) | 0.0 (0.0, 6.6) | 0.0 (0.0, 16.1) | 0.0 (0.0, 16.8) | |
| Functional constipation | 7.0 (3.4, 12.5) | 2.3 (0.1, 12.0) | 9.1 (3.0, 20.0) | 12.5 (2.7, 32.4) | 5.0 (0.1, 24.9) | |
| Functional diarrhea | 7.8 (4.0, 13.5) | 4.5 (0.6, 15.5) | 7.4 (2.1, 17.9) | 8.7 (1.1, 28.0) | 15.0 (3.2, 37.9) | |
| Functional abdominal bloating/distention | 5.5 (1.5, 13.4) | 4.3 (0.1, 21.9) | 11.5 (2.4, 30.2) | 0.0 (0.0, 28.5) | 0.0 (0.0, 24.7) | |
| *Anorectal disorders* | 14.1 (8.8, 20.9) | 15.9 (6.6, 30.1) | 9.1 (3.0, 20.0) | 17.4 (5.0, 38.8) | 20.0 (5.7, 43.7) | |
| Fecal incontinence | 14.1 (8.8, 20.9) | 15.9 (6.6, 30.1) | 9.1 (3.0, 20.0) | 17.4 (5.0, 38.8) | 20.0 (5.7, 43.7) | |
| Functional anorectal pain | 0.0 (0.0, 2.6) | 0.0 (0.0, 8.0) | 0.0 (0.0, 6.5) | 0.0 (0.0, 14.8) | 0.0 (0.0, 16.8) | |

NOTE: MT: medical treatment, RYGB: Roux-en-Y gastric bypass, SG: sleeve gastrectomy, IBS-C: irritable bowel syndrome with predominant constipation, IBS-D: irritable bowel syndrome with predominant diarrhea, IBS-M: irritable bowel syndrome with mixed bowel habits, IBS-U: irritable bowel syndrome unsubtyped.
