# Supplementary material for: Prevalence and Factors Associated With Symptom Profiles of Disorders of Gut‐Brain Interaction in Obesity Before and After Treatment
Source: Neurogastroenterol Motil. 2025 Mar 10;38:e70017. doi: 10.1111/nmo.70017 (PMC13121869; doi:10.1111/nmo.70017)
Supplement: Supplementary file 6 — Table S3. [file NMO-38-e70017-s002.docx]

**Supplementary table 4.** Proportion of patients with one, two, and three/four DGBI at baseline in diabetes only patients (n=144)

| **Number of DGBI regions affected** | **Diabetes with comorbid DGBI (n=83) (%)** |
| --- | --- |
| One | 57.8 (46.5, 68.6) |
| Two | 26.5 (17.4, 37.3) |
| Three or four | 15.7 (8.6, 25.3) |

NOTE: DGBI: disorder of gut-brain interaction
