# Supplementary material for: Prevalence and Factors Associated With Symptom Profiles of Disorders of Gut‐Brain Interaction in Obesity Before and After Treatment
Source: Neurogastroenterol Motil. 2025 Mar 10;38:e70017. doi: 10.1111/nmo.70017 (PMC13121869; doi:10.1111/nmo.70017)
Supplement: Supplementary file 7 — Table S4. [file NMO-38-e70017-s008.docx]

**Supplementary table 3.** Comparison of baseline health outcomes in patients with vs. without a DGBI at baseline in diabetes only patients (n=144)

|  | **Diabetes only cohort (n=144)** | | |
| --- | --- | --- | --- |
|  | **With DGBI (n=573)** | **Without DGBI (n=366)** | **p-value** |
| Gender (% female) | 63.9 (52.6, 74.1) | 62.3 (49.0, 74.4) | 0.85 |
| Gender (% male) | 36.1 (25.9, 47.4) | 37.7 (25.6, 51.0) | 0.85 |
| Class I obesity (%) | 5.1 (1.4, 12.5) | 3.4 (0.4, 11.7) | 0.63 |
| Class II obesity (%) | 57.0 (45.3, 68.1) | 37.3 (25.0, 50.9) | **0.02** |
| Class III obesity (%) | 38.0 (27.3, 49.6) | 59.3 (45.7, 71.9) | **0.01** |
| BMI (kg/m^2^) | 40.42 ± 4.84 | 41.72 ± 5.16 | 0.13 |
| HbA1c  (mmol/mol) | 56.06 ± 15.41 | 56.06 ± 14.04 | 0.99 |
| Glucose (mmol/L) | 9.36 ± 2.86 | 9.59 ± 3.10 | 0.67 |
| LDL-P cholesterol (nmol/L) | 2.60 ± 0.74 | 2.48 ± 0.79 | 0.37 |
| HDL-P cholesterol (nmol/L- | 1.11 ± 0.25 | 1.16 ± 0.31 | 0.37 |
| Triglycerides (mmol/L) | 2.25 ± 1.11 | 2.02 ± 1.06 | 0.25 |
| Anxiety (BAI score) | 8.00 [11.00] | 7.00 [10.00] | 0.40 |
| Depression (PHQ-9 score) | 5.00 [6.00] | 4.00 [5.75] | 0.17 |
| QoL (EQ5D index score) | 0.69 ± 0.16 | 0.69 ± 0.19 | 0.81 |

NOTE: DGBI: disorder of gut-brain interaction, HbA1c: Hemoglobin A1C, LDL-P low density lipoprotein particles, HDL-P: high density lipoprotein particles, QoL: quality of life
