# Supplementary material for: Prevalence and Factors Associated With Symptom Profiles of Disorders of Gut‐Brain Interaction in Obesity Before and After Treatment
Source: Neurogastroenterol Motil. 2025 Mar 10;38:e70017. doi: 10.1111/nmo.70017 (PMC13121869; doi:10.1111/nmo.70017)
Supplement: Supplementary file 8 — Table S5. [file NMO-38-e70017-s007.docx]

**Supplementary table 5.** Prevalence of DGBI diagnoses at baseline and follow-up stratified by different treatments, diabetes only (n=95)

| ***Diagnosis*** | **Baseline** | | | | **Follow-up at 2 years** | | | |
| --- | --- | --- | --- | --- | --- | --- | --- | --- |
|  | **Overall (n=95)** | **MT (n=30)** | **RYGB (n=44)** | **SG (n=21)** | **Overall (n=95)** | **MT (n=30)** | **RYGB (n=44)** | **SG (n=21)** |
| *Esophageal disorders* | 23.2 (15.1, 32.9) | 30.0 (14.7, 49.4) | 22.7 (11.5, 37.8) | 14.3 (3.0, 36.6) | 15.6 (8.8, 24.7) | 7.1 (0.9, 23.5) | 20.9 (10.0, 36.0) | 15.8 (3.4, 39.6) |
| Functional chest pain | 4.3 (1.6, 9.0) | 13.8 (3.9, 31.7) | 2.3 (0.1, 12.3) | 0.0 (0.0, 16.1) | 3.3 (0.7, 9.4) | 3.6 (0.1, 18.3) | 2.3 (0.1, 12.3) | 5.3 (0.1, 26.0) |
| Functional heartburn | 5.4 (1.8, 12.1) | 13.3 (3.8, 30.7) | 13.6 (5.2, 27.4) | 4.8 (0.1, 23.8) | 4.4 (1.2, 11.0) | 3.6 (0.1, 18.3) | 4.7 (0.6, 15.8) | 5.3 (0.1, 26.0) |
| Globus | 3.5 (0.7, 9.9) | 0.0 (0.0, 11.9) | 2.7 (0.1, 14.2) | 10.0 (1.2, 31.7) | 0.0 (0.0, 4.1) | 0.0 (0.0, 12.3) | 0.0 (0.0, 8.6) | 0.0 (0.0, 17.6) |
| Functional dysphagia | 6.4 (2.4, 13.4) | 6.7 (0.8, 22.1) | 7.0 (1.5, 19.1) | 4.8 (0.1, 23.8) | 7.9 (3.2, 15.5) | 0.0 (0.0, 12.3) | 14.3 (5.4, 28.5) | 5.3 (0.1, 26.0) |
| *Gastroduodenal disorders* | 23.2 (15.1, 32.9) | 33.3 (17.3, 52.8) | 18.2 (8.2, 32.7) | 19.0 (5.4, 41.9) | 19.8 (12.2, 29.4) | 10.7 (2.3, 28.2) | 22.7 (11.5, 37.8) | 26.3 (9.1, 51.2) |
| Functional dyspepsia | 10.6 (5.2, 18.7) | 26.7 (12.3, 45.9) | 2.3 (0.1, 12.3) | 4.8 (0.1, 23.8) | 9.1 (4.0, 17.1) | 0.0 (0.0, 12.8) | 11.9 (4.0, 25.6) | 15.8 (3.4, 39.6) |
| Belching disorder | 7.5 (3.1, 14.9) | 10.0 (2.1, 26.5) | 7.1 (1.5, 19.5) | 4.8 (1.0, 23.8) | 7.8 (3.2, 15.4) | 7.4 (0.9, 24.3) | 6.8 (1.4, 18.7) | 10.5 (1.3, 33.1) |
| Nausea and vomiting disorders | 13.7 (7.5, 22.3) | 16.7 (5.6, 34.7) | 13.6 (5.2, 27.4) | 9.35(1.2, 30.4) | 12.1 (6.2, 20.6) | 7.1 (0.9, 23.5) | 15.9 (6.6, 30.1) | 10.5 (1.3, 33.1) |
| Rumination syndrome | 0.0 (0.0, 3.8) | 0.0 (0.0, 11.6) | 0.0 (0.0, 8.0) | 0.0 (0.0, 16.1) | 2.2 (0.3, 7.8) | 0.0 (0.0, 12.8) | 2.3 (0.1, 12.0) | 5.3 (0.1, 26.0) |
| *Bowel disorders* | 40.0 (30.1, 50.6) | 33.3 (17.3, 52.8) | 40.9 (26.3, 56.8) | 47.6 (25.7, 70.2) | 37.2 (27.5, 47.8) | 48.3 (29.4, 67.5) | 29.5 (16.8, 45.2) | 38.1 (18.1, 61.6) |
| Irritable bowel syndrome | 24.2 (16.0, 34.1) | 23.3 (9.9, 42.3) | 22.7 (11.5, 37.8) | 28.6 (11.3, 52.2) | 14.9 (8.4, 23.7) | 20.7 (8.0, 39.7) | 9.1 (2.5, 21.7) | 19.0 (5.4, 41.9) |
| IBS-C | 2.2 (0.3, 7.6) | 0.0 (0.0, 11.6) | 2.3 (0.1, 12.0) | 5.3 (0.1, 26.0) | 3.4 (0.7, 9.5) | 3.8 (0.1, 19.6) | 2.3 (0.1, 12.0) | 5.3 (0.1, 26.0) |
| IBS-D | 10.8 (5.3, 18.9) | 13.3 (3.8, 30.7) | 9.1 (2.5, 21.7) | 10.5 (1.3, 33.1) | 4.5 (1.2, 11.1) | 7.7 (0.9, 25.1) | 2.3 (0.1, 12.0) | 5.3 (0.1, 26.0) |
| IBS-M | 9.7 (4.5, 17.6) | 6.7 (0.8, 22.1) | 11.4 (3.8, 24.6) | 10.5 (1.3, 33.1) | 6.7 (2.5, 14.1) | 11.5 (2.4, 30.2) | 2.3 (0.1, 12.0) | 10.5 (1.3, 33.1) |
| IBS-U | 1.1 (0.0, 5.8) | 3.3 (0.1, 17.2) | 0.0 (0.0, 8.0) | 0.0 (0.0, 17.6) | 1.1 (0.0, 6.1) | 0.0 (0.0, 13.2) | 2.3 (0.1, 12.0) | 0.0 (0.0, 17.6) |
| Functional constipation | 6.3 (2.4, 13.2) | 3.3 (0.1, 17.2) | 6.8 (1.4, 18.7) | 9.5 (1.2, 30.4) | 18.1 (10.9, 27.4) | 20.7 (8.0, 39.7) | 15.9 (6.6, 30.1) | 19.0 (5.4, 41.9) |
| Functional diarrhea | 5.3 (1.7, 11.9) | 3.3 (0.1, 17.2) | 4.5 (0.6, 15.5) | 9.5 (1.2, 30.4) | 0.0 (0.0, 3.8) | 0.0 (0.0, 11.9) | 0.0 (0.0, 8.0) | 0.0 (0.0, 16.1) |
| Functional abdominal bloating/distention | 8.2 (2.3, 19.6) | 5.6 (0.1, 27.3) | 14.3 (3.0, 36.3) | 0.0 (0.0, 30.4) | 5.5 (1.5, 13.4) | 10.5 (1.3, 33.1) | 5.6 (0.7, 18.7) | 0.0 (0.0, 18.5) |
| *Anorectal disorders* | 13.8 (7.6, 22.5) | 20.0 (7.7, 38.6) | 6.8 (1.4, 18.7) | 20.0 (5.7, 43.7) | 13.5 (7.2, 22.4) | 11.5 (2.4, 30.2) | 13.6 (5.2, 27.4) | 15.8 (3.4, 39.6) |
| Fecal incontinence | 13.8 (7.6, 22.5) | 20.0 (7.7, 38.6) | 6.8 (1.4, 18.7) | 20.0 (5.7, 43.7) | 10.2 (4.8, 18.5) | 4.0 (0.1, 20.4) | 11.4 (3.8, 24.6) | 15.8 (3.4, 39.6) |
| Functional anorectal pain | 0.0 (0.0, 3.8) | 0.0 (0.0, 11.6) | 0.0 (0.0, 8.0) | 0.0 (0.0, 16.8) | 3.4 (0.7, 9.5) | 7.7 (0.9, 25.1) | 2.3 (0.1, 12.0) | 0.0 (0.0, 17.6) |

NOTE: All patients who were not appointed to a treatment group (n=20) or who were lost in follow-up (additional n=29) (total n=49) were not considered in this analysis. MT: medical treatment, RYGB: Roux-en-Y gastric bypass, SG: sleeve gastrectomy, IBS-C: irritable bowel syndrome with predominant constipation, IBS-D: irritable bowel syndrome with predominant diarrhea, IBS-M: irritable bowel syndrome with mixed bowel habits, IBS-U: irritable bowel syndrome unsubtyped.
